# Clinicopathological study of oral focal mucinosis: a retrospective case series

**DOI:** 10.4317/medoral.22291

**Published:** 2018-06-21

**Authors:** Amanda-Katarinny-Goes Gonzaga, Denise-Hélen-Imaculada-Pereira de Oliveira, Maria-Luiza-Diniz-de Sousa Lopes, Tiago-João-da Silva Filho, Lélia-Maria-Guedes Queiroz, Éricka-Janine-Dantas da Silveira

**Affiliations:** 1 DDS, MSc, PhD student, Postgraduate Program in Oral Pathology, Department of Dentistry, Federal University of Rio Grande do Norte, Natal, RN, Brazil. Av. Senador Salgado Filho, 1787, Lagoa Nova, CEP 59056-000 Natal, RN, Brazil; 2 DDS, MSc, PhD, Professor, Department of Dentistry, Federal University of Ceará, Sobral, CE, Brazil. Rua Estanislau Frota, S/N, Centro, CEP 62010-560, Sobral, CE, Brazil; 3 DDS, MSc, PhD, Postgraduate Program in Oral Pathology, Department of Dentistry, Federal University of Rio Grande do Norte, Natal, RN, Brazil. Av. Senador Salgado Filho, 1787, Lagoa Nova, CEP 59056-000 Natal, RN, Brazil; 4 DDS, MSc, PhD, Professor, Department of Dentistry, State University of Paraíba, Campina Grande, PB, Brazil. Rua Baraúnas, 351, Bairro Universitário, CEP 58429-500, Campina Grande, PB, Brazil; 5 DDS, MSc, PhD, Professor, Postgraduate Program in Oral Pathology, Department of Dentistry, Federal University of Rio Grande do Norte, Natal, RN, Brazil. Av. Senador Salgado Filho, 1787, Lagoa Nova, CEP 59056-000 Natal, RN, Brazil

## Abstract

**Background:**

Oral focal mucinosis (OFM) is a rare soft tissue lesion of unknown etiology that exhibits tumor-like growth. It is considered the oral counterpart of cutaneous focal mucinosis or cutaneous myxoid cyst. This is a retrospective study of oral OFM diagnosed over a period of 42 years at an oral pathology service.

**Material and Methods:**

Clinical, histopathological and immunohistochemical data were analyzed. Alcian blue staining and S-100 immunohistochemistry were performed.

**Results:**

Eleven cases were retrieved (4:1 female-to-male ratio). The mean age was 44 years. The gingiva was the most affected site. The main clinical presentation was sessile or pedunculated lesions of fibrous or hyperplasic appearance, most of them asymptomatic. Positive Alcian blue staining and absence of S-100 protein were observed in all specimens, which supported the histological diagnosis of OFM. Surgical excision was the treatment of choice.

**Conclusions:**

Although rare, this study supports the inclusion of OFM in the differential diagnosis of intraoral myxoid lesions.

** Key words:**Oral focal mucinosis, myxomatous lesion, connective tissue diseases, diagnosis.

## Introduction

Oral focal mucinosis (OFM) is a rare soft tissue lesion of unknown etiology that exhibits tumor-like growth. It is considered the oral counterpart of cutaneous focal mucinosis or cutaneous myxoid cyst. OFM was first described by Tomich in 1974 as the consequence of local hyaluronic acid overproduction by fibroblasts, and few cases have been reported since then (1). Although OFM can affect different sites in the oral cavity, it is most commonly found on the gingiva and presents as a painless, sessile or pedunculated mass of the same color as the surrounding mucosa (2). Histologically, OFM is characterized by a well-circumscribed area of myxomatous connective tissue containing mucinous material, surrounded by denser collagenous connective tissue (3,4-7).

The aim of this study was to retrospectively analyze the clinical and histological data of 11 OFM cases diagnosed at a referral service for Oral Pathology. In addition, we discuss some important aspects regarding the diagnosis of this uncommon lesion.

## Material and Methods

After approval by the Ethics Committee (1.579.149/2016), all cases diagnosed as OFM between 1974 and 2016 were reviewed. Data such as age and sex of the patient, anatomical site, tumor progression, and symptoms were obtained from the biopsy records.

For histopathological analysis, all 5-μm thick sections stained with hematoxylin and eosin were re-evaluated by two oral pathologists under a light microscope (Olympus CX31; Olympus Japan Co., Tokyo, Japan). In addition, Alcian blue staining (pH 2.5) was used for the identification of mucosubstances. For immunohistochemistry, all OFM specimens were fixed in formalin and embedded in paraffin. Three-μm thick tissue sections were obtained and mounted on silanized glass slides (3-aminopropyltriethoxysilane; Sigma Chemical Co., St. Louis, MO, USA). Immunohistochemical analysis was performed using the streptavidin-biotin complex method and the slides were incubated overnight with anti-S-100 antibody as primary antibody (Dako, Rocklin, CA, USA) diluted 1: 200. Oral neurofibroma specimens were used as positive control.

## Results

Among the 14,204 oral lesion biopsies obtained over a period of 42 years and retrieved from the archives of the Oral Pathology service, 11 cases (0.07%) were diagnosed as OFM. Table 1 shows the clinical, pathological, and staining information of the cases. Nine of the 11 patients were female and two were male. The mean age at the time of diagnosis was 43.9 years (range: 23 to 78). Information about lesion location was available in 10 cases. There was a predilection for the gingiva (n=4), followed by the alveolar ridge (n=3) and mucosa of the hard palate (n=3). The duration of the lesions ranged from 2 to 36 months.

Clinically, the lesions appeared as hyperplastic (n=2), tumoral (n=2), papillomatous (n=1), and fibrous (n=1). The shape was pedunculated in four cases and no associated symptoms were observed in six. This information was not available in the remaining cases. All lesions had a normal color and most of them had a soft consistency. The size of the lesions ranged from 0.25 to 1.2 cm. Tooth displacement was observed in two cases (cases 6 and 7).

**Figure 1:**
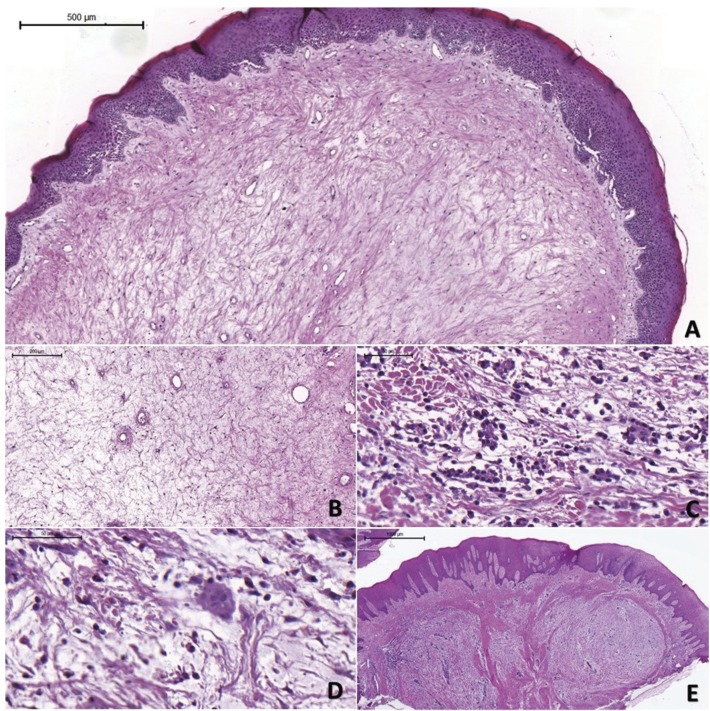
Histopathological findings of the OFMs. (A) Photomicrography of OFM revealing myxoid area surrounded by dense connective tissue covered with squamous epithelium. (B) Myxoid area in detail showing delicate collagen fibrils separated by mucinous material. (C) OFM revealing moderate inflammatory infiltrate associated with Russel bodies. (D) Multinucleated giant cells were observed in one case, which also showed inflammation. (E) OFM showing an uncommon multinodular pattern (Hematoxylin-eosin).

The main clinical diagnoses were oral traumatic fibroma, followed by peripheral giant cell granuloma. Other hypotheses included fibrotic hyperplasia, pyogenic granuloma, granulation tissue, gingival hyperplasia, peripheral odontogenic fibroma, central giant cell granuloma, pleomorphic adenoma, and papilloma ( Table 1). Histologically, all lesions exhibited well-circumscribed myxoid connective tissue surrounded by a band of dense connective tissue of variable thickness beneath the epithelium (Fig. 1A). The myxomatous areas were palely stained and composed of loose connective tissue containing very thin, scattered and randomly arranged collagen fiber bundles, several fibroblasts of variable shape (spindle, stellate, and round), and small-diameter blood vessels (Fig. 1B).

**Table 1:**
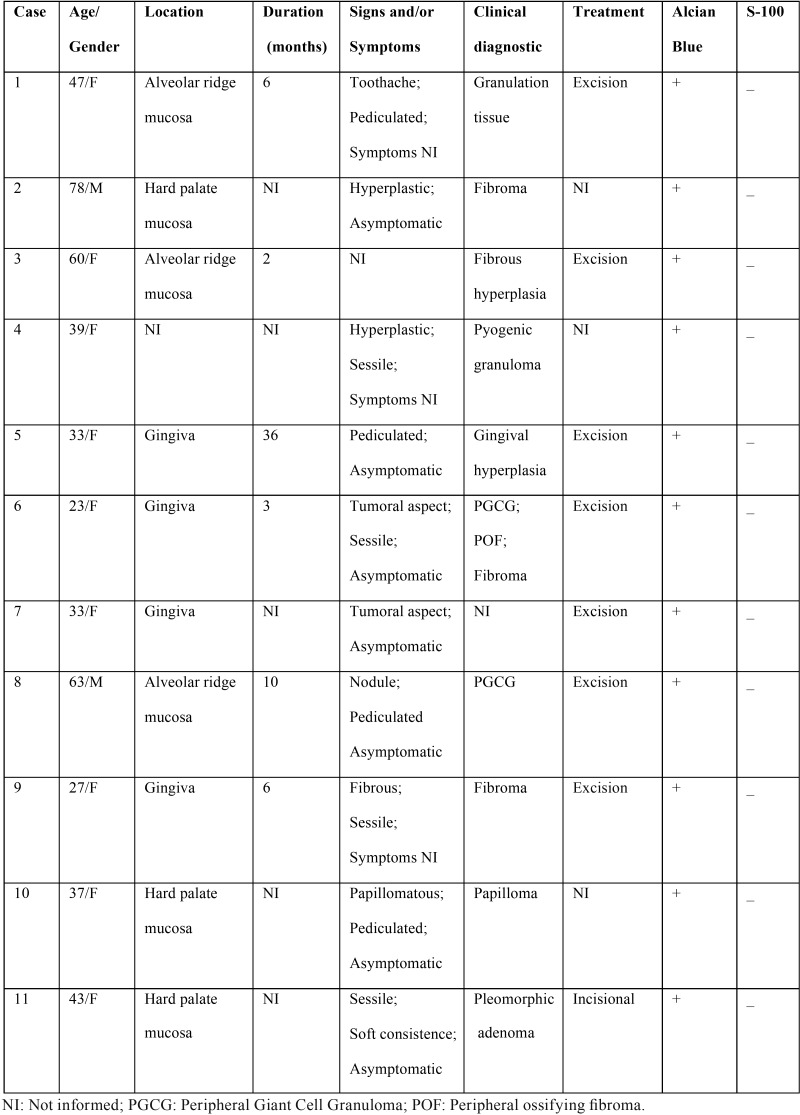
Demographic, clinical, histochemical and immunohistochemical features of 10 cases of oral focal mucinosis.

Occasional findings were focal areas of a lymphocytic inflammatory infiltrate in two cases, including Russel bodies in one of them (Fig. 1C). Hemosiderin pigmentation was observed in two cases and multinucleated giant cells in one (Fig. 1D). There was a particular case of a multinodular pattern with myxoid central areas surrounded by thin collagen fiber bundles (Fig. 1E).

**Figure 2:**
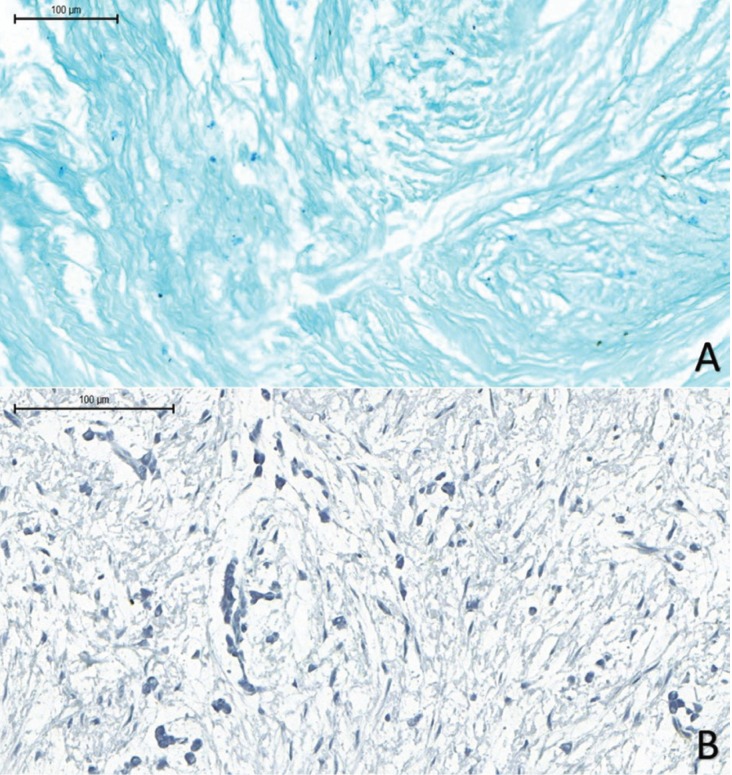
(A) Hyaluronic acid (alcianophilic material) in the myxomatous tissue (Alcian blue, pH 2.5). (B) Negative immunohistochemical reaction for S-100 protein in the myxoid tissue (HIDEF; Scale Bar100μm).

Histochemical staining with Alcian blue was positive (Fig. 2A) and immunohistochemical staining with anti-S-100 antibody was negative in all cases (Fig. 2B).

## Discussion

The pathogenesis of OFM involves the overproduction of hyaluronic acid by fibroblasts and its consequent accumulation among collagen fibers (1,8,9), but the etiology of the disease remains largely unknown (2,6,8-10). According to some authors, localized inflammation associated or not with local trauma cannot be excluded as a potential etiological factor of OFM (5,7,11,12), but the topic remains controversial.

Slightly more than 50 cases of OFM have been reported in the English literature, most of them affecting women in the fourth or fifth decade of life (7,11). There is a gingival site predilection, followed by the mucosa of the hard palate (6,8). In the present case series, there was a female-to-male ratio of 4:1 and predilection of OFM for the gingiva, but individuals of a wide age range were affected.

A preoperative clinical diagnosis of OFM is unlikely because of the lack of any pathognomonic clinical feature. Furthermore, OFM may be easily mistaken for fibroma, pyogenic granuloma, peripheral ossifying fibroma, mucocele, or similar lesions (5,6,8,9). According to previous reports, OFM presents as a painless, sessile, nodular mass of the same color as the adjacent mucosa (2,5,9,10). The surface is typically smooth and non-ulcerated (2,9). Ulceration is rare and may be due to secondary trauma (12). Most of the present cases were asymptomatic, sessile or pedunculated lesions of fibrous or hyperplastic appearance, in agreement with the literature. Tooth displacement was observed in two cases. One of these cases (case 6) has been previously reported to exhibit a particularly large OFM (9), while no information about the size of the lesion was available for the other case (case 7).

Given the rarity of OFM and its clinical similarity to several inflammatory reactional lesions, the preoperative diagnosis is almost impossible (1,5,8,9). The provisional clinical diagnoses observed in the present study, which included many reactional lesions while OFM was never mentioned, support this fact.

Histopathological analysis of OFM reveals a myxoid tissue area surrounded by dense collagenous connective tissue (2,6,11). Loosely arranged collagen fibrils permeate the myxoid area, which are widely separated from one another by overproduction of hyaluronic acid and interspersed with fibroblasts of variable morphology (9,11). Inflammatory infiltration may occur but is uncommon (2), as observed in our cases.

Soft-tissue myxoma, nerve sheath myxoma, inflammatory fibroepithelial hyperplasia with myxoid degeneration, and odontogenic myxoma are the main histological differential diagnoses of OFM (7,8). According to Tomich (1), the distinction between OFM and lesions with myxomatous changes can be easily made by careful morphological examination, considering that in the latter the myxoid area usually blends into the surrounding connective tissue, while OFG exhibits a very well-defined myxoid area. Histochemical staining with Alcian blue is strong and diffuse in OFM, which confirms the presence of abundant mucin dispersed in connective tissue (1,2,11,12). Additional immunohistochemistry for S-100 protein also assists the diagnosis since staining for S-100 is positive in myxoid neural lesions, such as nerve sheath myxoma, and negative in OFM (2,9). In the present case series, the morphological, histochemical and immunohistochemical findings observed meet the diagnostic criteria for OFM.

Surgical excision is the recommended treatment for OFM and recurrence is rare (2,6,9). The present study confirms OFM as an uncommon lesion that mainly affects the gingiva of adult women. Histological analysis and special stains, such as Alcian blue and S-100, are important for the correct diagnosis. Although rare, OFM should be included in the differential diagnosis of oral soft tissue lesions, particularly lesions located in the gingiva.
